# Establishment of an Autophagy-Related Clinical Prognosis Model for Predicting the Overall Survival of Osteosarcoma

**DOI:** 10.1155/2021/5428425

**Published:** 2021-09-22

**Authors:** Jianyi Li, Xiaojie Tang, Yukun Du, Jun Dong, Zheng Zhao, Huiqiang Hu, Tao Song, Jianwei Guo, Yan Wang, Tongshuai Xu, Cheng Shao, Yingyi Sheng, Yongming Xi

**Affiliations:** ^1^Department of Orthopaedic Surgery, The Affiliated Hospital of Qingdao University, Qingdao 266071, China; ^2^Yantai Affiliated Hospital of Binzhou Medical University, 264010 Yantai, China; ^3^Department of Spinal Surgery, Shandong Provincial Hospital Affiliated to Shandong University, Jinan, Shandong 250021, China

## Abstract

**Purpose:**

Osteosarcoma is the most common primary and highly invasive bone tumor in children and adolescents. The purpose of this study is to construct a multi-gene expression feature related to autophagy, which can be used to predict the prognosis of patients with osteosarcoma. *Materials and methods.* The clinical and gene expression data of patients with osteosarcoma were obtained from the target database. Enrichment analysis of autophagy-related genes related to overall survival (OS-related ARGs) screened by univariate Cox regression was used to determine OS-related ARGs function and signal pathway. In addition, the selected OS-related ARGs were incorporated into multivariate Cox regression to construct prognostic signature for the overall survival (OS) of osteosarcoma. Use the dataset obtained from the GEO database to verify the signature. Besides, gene set enrichment analysis (GSEA) were applied to further elucidate the molecular mechanisms. Finally, the nomogram is established by combining the risk signature with the clinical characteristics.

**Results:**

Our study eventually included 85 patients. Survival analysis showed that patients with low riskScore had better OS. In addition, 16 genes were included in OS-related ARGs. We also generate a prognosis signature based on two OS-related ARGs. The signature can significantly divide patients into low-risk groups and high-risk groups, and has been verified in the data set of GEO. Subsequently, the riskScore, primary tumor site and metastasis status were identified as independent prognostic factors for OS and a nomogram were generated. The C-index of nomogram is 0.789 (95% CI: 0.703~0.875), ROC curve and calibration chart shows that nomogram has a good consistency between prediction and observation of patients.

**Conclusions:**

ARGs was related to the prognosis of osteosarcoma and can be used as a biomarker of prognosis in patients with osteosarcoma. Nomogram can be used to predict OS of patients and improve treatment strategies.

## 1. Introduction

Osteosarcoma is the most common primary and highly invasive bone tumor, which originates from primitive mesenchymal cells and usually occurs in children and adolescents [1–3]. Although great progress has been made in treatment methods including surgery, chemotherapy and neoadjuvant chemotherapy, there are still a considerable number of relapses or metastases [4, 5]. The 5-year survival rate of children and young people with non-metastatic osteosarcoma was 60%. However, for patients with metastasis or poor response to initial treatment, the 5-year survival rate was only 20% [6, 7]. A better understanding of the pathogenesis of osteosarcoma and identification of biomarkers that can predict the prognosis of osteosarcoma are essential for improving the prognosis of patients. Therefore, there is an urgent need for a more accurate quantitative prediction tool to assist clinical diagnosis and treatment.

Autophagy is a catabolism process in which damaged organelles and macromolecules are degraded and circulated in cells [8]. Since Christian de Duve proposed the term autophagy in 1963 [9, 10], great progress has been made in using this approach to improve clinical outcomes, especially in cancer patients. Recent studies have confirmed that autophagy is involved in many physiological and pathological regulation processes in vivo [8, 11–15], such as cell differentiation and development, cell abnormal structure degradation, immune stress, tumor inhibition, anti-aging, cell death and so on. The relationship between autophagy and tumorigenesis is a double-edged sword. In some cases, autophagy can inhibit tumorigenesis, but in most cases, autophagy promotes tumorigenesis [16]. It has been reported that there is a close relationship between autophagy and osteosarcoma，and autophagy-related genes (ARGs) may have potential application value as a prognostic biological marker for osteosarcoma patients. For example, Akin D et al. [17] found that tumor inhibition is caused by the decrease of ATG4B activity; Besides, Zhao et al. [18] showed that the positive expression of ATG5 related to TSSC3 may be a favorable prognostic indicator of osteosarcoma. It is worth noting that although several studies have explored the role of autophagy in the occurrence and development of osteosarcoma, most studies have focused on evaluating the relationship between a single autophagy gene and osteosarcoma, while there are few reports on a comprehensive understanding of the relationship between ARGs and the prognosis of osteosarcoma.

Therefore, in this study, we analyzed the gene expression data of osteosarcoma patients in the TARGET database, extracted ARGs related to the prognosis of osteosarcoma, and used to predict the prognosis of osteosarcoma and give recommended treatment targets. In addition, we developed a new prognosis model of osteosarcoma by combining the risk signature of ARGs and clinical parameters (primary tumor site, metastasis status). This model has a more accurate predictive ability than a single clinical risk factor.

## 2. Materials and Methods

### 2.1. Data Acquisition and Collation

The transcriptome and clinical data of 88 osteosarcoma samples were obtained from the TARGET Database (https://ocg.cancer.gov/programs/target/data-matrix，Date of data acquisition: 31 November 2019). The list of ARGs was extracted from the human autophagy database (HADb, http://www.autophagy.lu/clustering/index.html). Meanwhile, the messenger RNA (mRNA) expression profiles and clinical data of GSE21257 cohort were obtained from the Gene Expression Omnibus database (https://www.ncbi.nlm.nih.gov/geo/) as an external verification cohort.

### 2.2. Functional Enrichment Analysis of Prognosis-Related Autophagy Genes

Functional enrichment analysis is a method to analyze gene expression information [19]. Enrichment analysis was completed by “cluster Profiler” package in R software, and univariate Cox regression was completed by “survival” package in R software. First of all, the OS-related ARGs and their gene expression related to the OS of patients with osteosarcoma were screened by the univariate Cox regression analysis. Then, after over-fitting was excluded by the least absolute shrinkage and selection operator (LASSO) regression model, We used Gene Ontology (GO) to analyze OS-related ARGs to obtain the biological function of these genes. Meanwhile, the Kyoto Encyclopedia of Genes and Genomes (KEGG) were used to enrich the related pathways of OS-related ARGS. The statistically significant threshold for enrichment was adjusted p-value (q value) <0.05.

### 2.3. Construction and Verification of OS-Related ARGs Signature for Osteosarcoma Patients

Based on the OS-related ARGs obtained by LASSO regression model, the OS-related ARGs were verified in the GSE21257 cohort, Only the genes that have been successfully verified are selected for further analysis. On the basis of gene verification, multivariate Cox regression analysis was carried out. Then, the risk signature was established based on the regression coefficients and gene expression derived from the multivariate Cox regression model in the training cohort. The formula for calculating the riskScore is: riskScore = (expr gen1 × Coef gen1) + (expr gen2 × coef gen2) + …. + (expr genen × coef genen). Meanwhlie, patients with osteosarcoma were divided into low-risk group and high-risk group according to the median riskScore. The “survival” package were used to generate time-varying receiver operating characteristic (ROC) curve and Kaplan-Meier survival curve to distinguish the difference between high risk group and low risk group. In addition, the riskScore of the patient in the verification cohort is calculated according to the aforementioned risk signature. The Kaplan-Meier survival curve and survival ROC curve to show the predictive ability of signature in the verification cohort.

### 2.4. GSEA between the High- and Low-Risk Group in the Training set

To further explore the molecular mechanism of prognostic gene signature in the training cohort. Gene set enrichment analysis (GSEA) was performed in javaGSEA v.4.1.0. |NES| >1, P <0.05 and FDR<0.05 were considered statistically significant.

### 2.5. Clinical Correlation Analysis of OS-Related ARGs

Clinical correlation analysis is to observe whether ARGs involved in model construction are related to clinical characteristics [20]. This is done using the “beeswarm” software package in the R software. The correlation between ARGs and clinical characteristics was obtained by the “t-test” on the ARGs and clinical characteristics that were screened for prognosis of osteosarcoma.

### 2.6. Establish and Evaluate ARGs-Clinical Nomogram in the Training Group

In order to improve the clinical application of risk signature, we included the clinical information of osteosarcoma patients in the training group. After univariate and multivariate Cox regression analysis, a nomogram [21], for predicting 1-, 3- and 5-year OS was developed through the “survival” package to determine the relationship between these factors and the OS of patients. The C-index (The higher the value of C-index, the more accurate it is to predict the prognosis), the ROC curve, and the calibration chart were used to evaluate the model.

### 2.7. Statistic Analysis

All statistical analyses, including univariate and multivariate Cox regression, ROC curve analysis and Kaplan-Meier survival analysis were performed using RStudio software (https://rstudio.com/products/rstudio/download/, version: 1.2.5033) and the corresponding R software package. The quantitative data were expressed as mean ± standard deviation (SD). The comparison between the two groups was performed by t-test, and Kruskal-Wallis H was used for comparison among groups, and Fisher exact test was used for classified variables. Except for the special instructions, all statistical tests were bilateral, P <0.05 was considered to be statistically significant.

## 3. Results

### 3.1. Data Pre-Processing

In this study, 88 samples of osteosarcoma patients downloaded from the TARGET database were identified and preliminarily screened. Cases with repetition, lack of necessary clinical survival information were excluded, and 85 samples of osteosarcoma patients were obtained as the training group. The demographic and clinicopathological statistics of the 85 samples are shown in Table 1，The clinical characteristics of 53 verification cohort samples obtained from the GSE21257 cohort are shown in Table S1.The classification of age is realized by the best cut-off value obtained by the X-tile software (version 3.6.1).

### 3.2. Functional Enrichment Analysis of 16 Prognosis-Related Autophagy Genes

**Figure 1:**
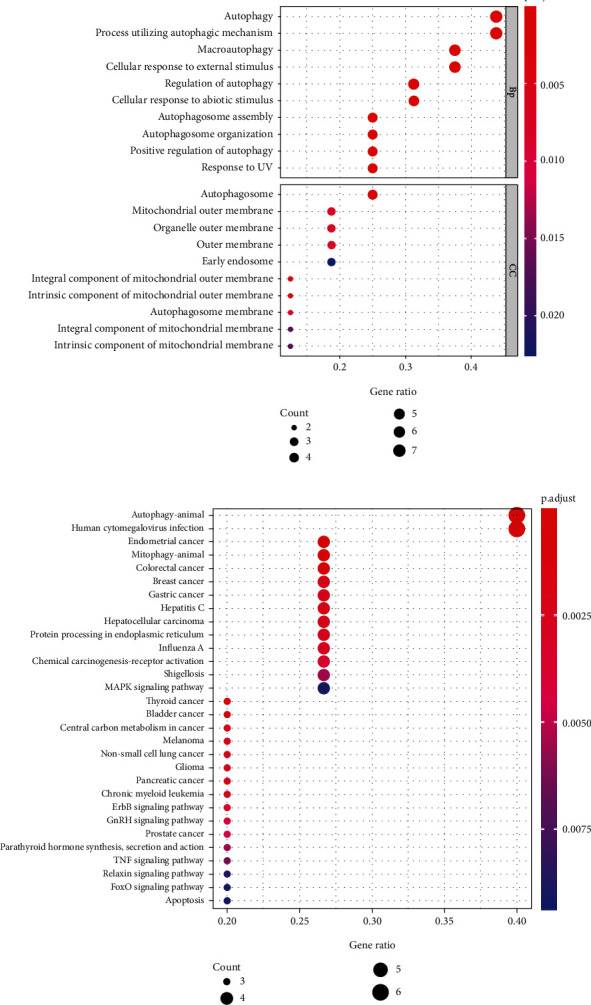
Enrichment analysis of ARGs related to the survival time of patients. (a) GO enrichment analysis showed the molecular function of ARGs. (b) KEGG enrichment analysis showed the signal pathway of ARGs. ARGs: Autophagy-related genes; GO: Gene Ontology; KEGG: Kyoto Encyclopedia of Genes and Genomes.

31 OS-related ARGs were screened by Univariate Cox regression from 85 osteosarcoma samples downloaded from the TARGET database and preprocessed (Supplementary file 1). The LASSO regression model was used to further eliminate the over-fitting, and 16 OS- related ARGs were obtained (Supplementary file 2, Figure S1). In order to understand the function of 16 OS-related ARGs of patients with osteosarcoma, we performed GO and KEGG analysis. Interestingly, we found that the results of GO analysis and KEGG analysis are mostly related to autophagy. GO analyses includes three types: Biological Process (BP), Cellular Component (CC) and Molecular Functional (MF). Of which, compared with the CC category, OS-related ARGs were significantly enriched in the BP category. In BP category, OS-related ARGs are significantly enriched in autophagy, a process utilizing autophagic mechanism, cellular response to external stimulus and macroautophagy; in CC category, OS-related ARGs are highly enriched in autophagosome (Figure 1(a)). In addition, KEGG pathway analysis showed that OS-related ARGs were significantly enriched in autophagy-animal pathway and Human cytomegalovirus infection (Figure 1(b)).

### 3.3. Construction and Verification of Risk Signature

Based on the above LASSO regression model screening of 16 OS-related ARGs, four OS-related ARGs were successfully verified in the GSE21257 dataset, which were ATG4A、MAPK1 and MYC, respectively (Supplementary file 3). Then, the expression of the four genes verified successfully was incorporated into the multivariate Cox regression model to screen and obtain the risk regression coefficient to establish the risk signature (Supplementary file 4). The riskScore of each sample was calculated as follows: riskScore = -1.155∗ATG4A+0.478∗MYC. According to the median riskScore of all samples, the samples were divided into a high-risk group and low-risk group.

**Figure 2:**
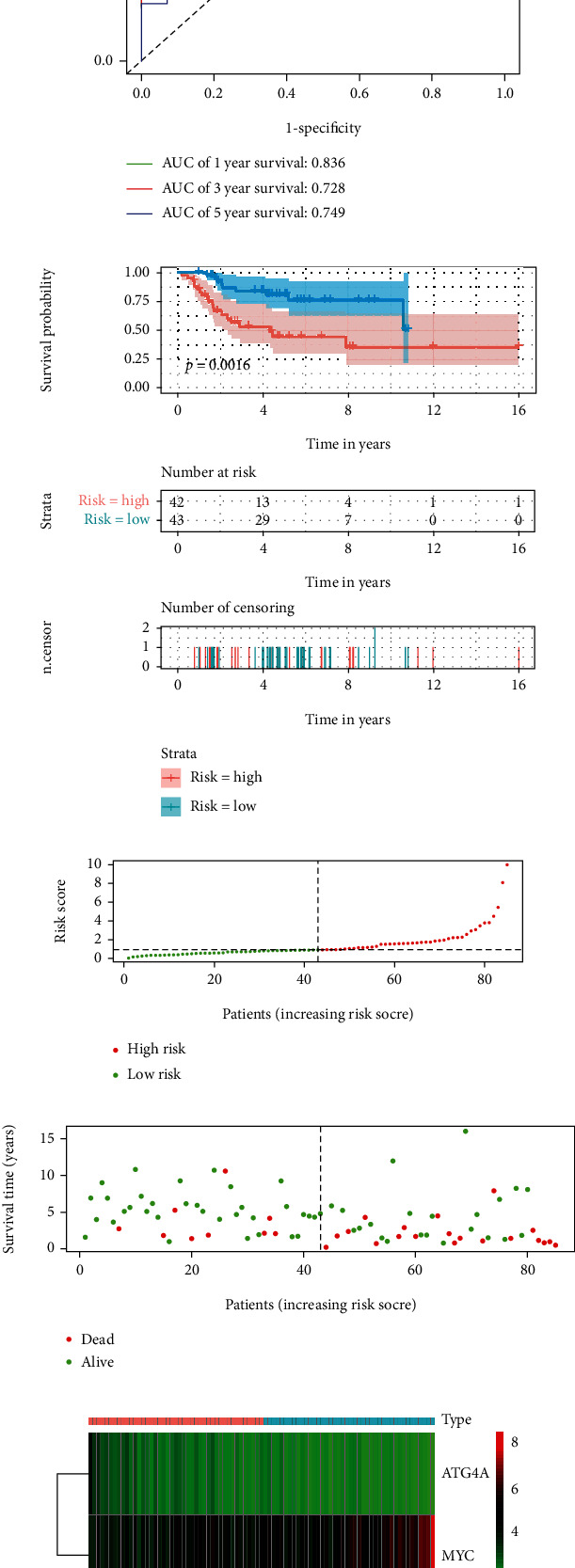
The construction of risk signature. A and B, ROC curve analysis and survival analysis of training cohort. ROC curve shows that the risk signature of training cohort can be used to predict the prognosis of patients with osteosarcoma. Kaplan-Meier survival curve shows that the risk signature of high-risk group and low-risk group of training cohort have predictive value for prognosis. C-E, Training cohort riskScore distribution, patient survival status distribution, and relationship between riskScore and expression of two OS-related ARGs. ROC: receiver operating characteristic; OS-related ARGs: autophagy-related genes related to overall survival.

**Figure 3:**
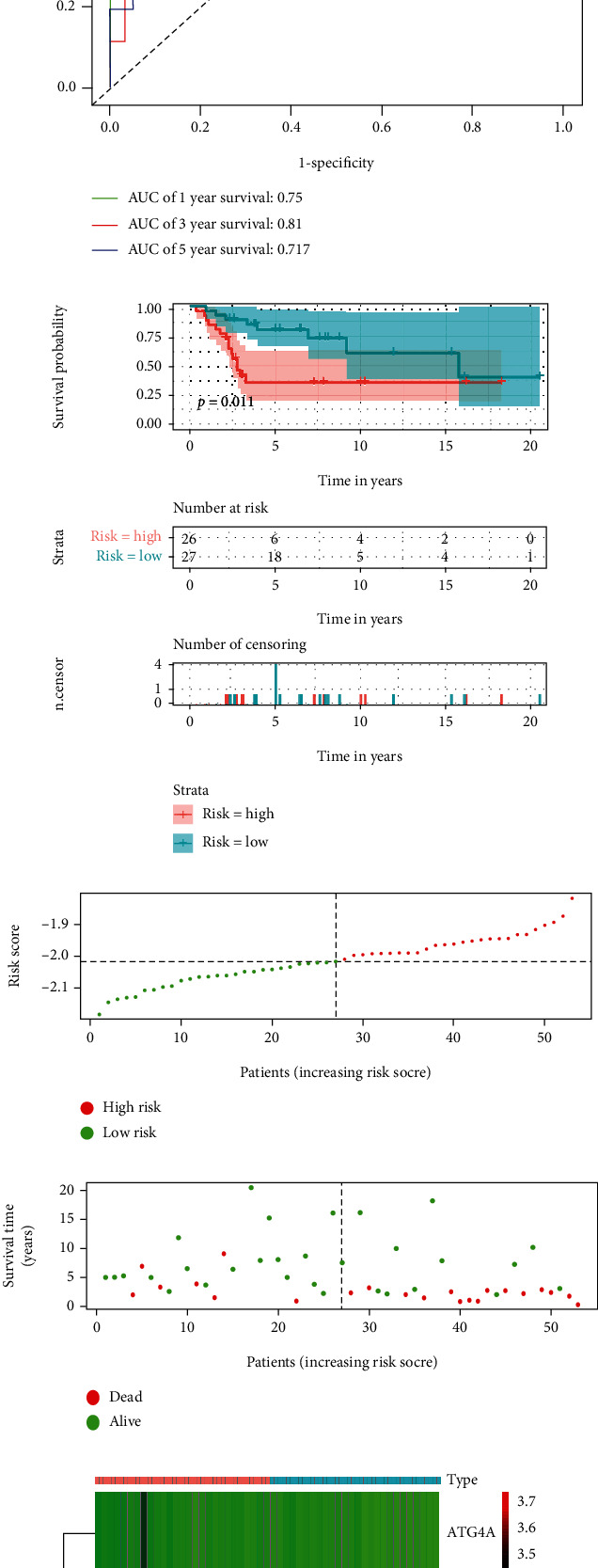
Verification of risk signature. (a), validation cohort ROC curve analysis, ROC curve showed that risk signature could be used to predict the prognosis of osteosarcoma patients; (b), validation cohort survival analysis, Kaplan-Meier survival curve shows that validation cohort high-risk group and low-risk group risk signature have predictive value for prognosis. C-E, Validation cohort riskScore distribution, patient survival status distribution, and relationship between riskScore and expression of two OS-related ARGs. ROC: receiver operating characteristic; OS-related ARGs: autophagy-related genes related to overall survival.

To verify the difference between the low-risk group and the high-risk group, we conducted ROC curve analysis and survival analysis (Figures 2(a), 2(b)), AUC values were 0.836, 0.728 and 0.749 in 1-, 3- and 5-year, respectively. The patient's survival curve showed that the OS of the high-risk group was significantly lower than that of the low-risk group. Generate risk score plots, survival status maps, and heat maps to show the difference between high-risk and low-risk patients (Figures 2(C)-2(e)). Subsequently, we verified the risk signature in an independent queue. The AUC value of the verification cohort was 0.75, 0.81 and 0.717 at 1-, 3- and 5-year, respectively (Figure 3(a)). The survival curve shows that patients in the low-risk group have a better prognosis than the high-risk group (Figure 3(b)). In addition, the risk score plots, survival status map and heat map show that there is a better distinction between the high-risk group and the low-risk group (Figures 3(C)-3(e)).

### 3.4. Gene Set Enrichment Analysis (GSEA)

**Figure 4:**
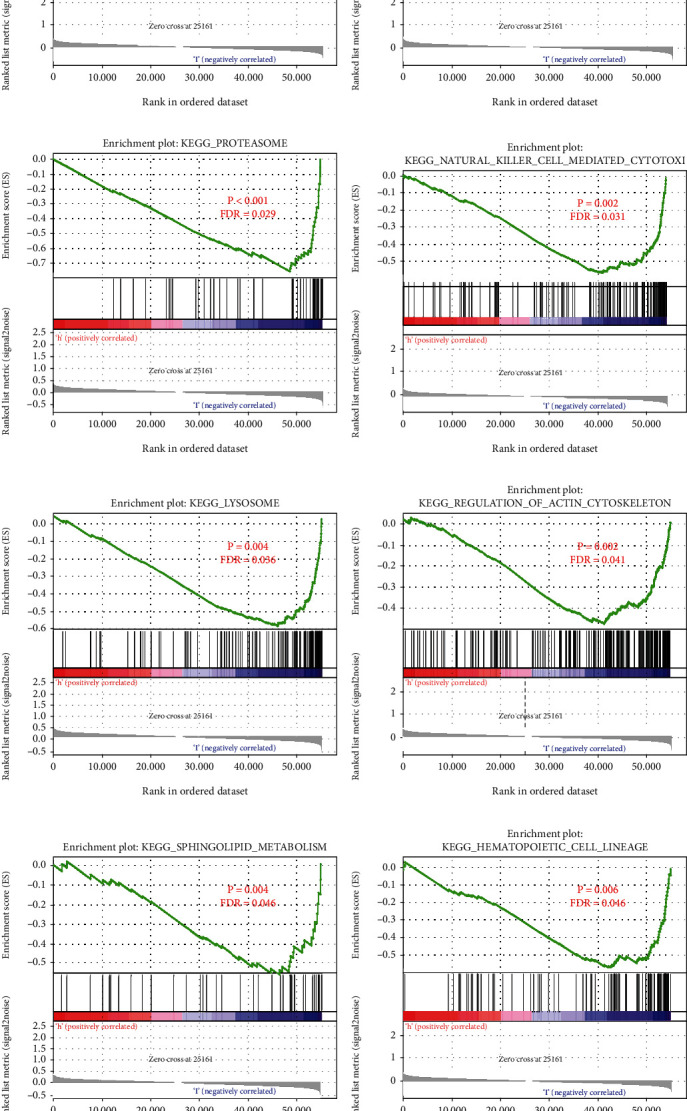
GSEA between the high- and low-risk group in the training set. A-L, The 12 KEGG pathways identified by GSEA were significantly enriched in the high-risk group. GSEA: Gene Set Enrichment Analysis.

In order to clarify the molecular mechanism of twoARGs in the training cohort, we performed GSEA based on the high- and low-risk group of ARGs (Figure 4). There were 12 KEGG pathways significant enrichment in the high risk group, including: T cell receptor signaling pathway, antigen processing and presentation, proteasome, natural killer cell mediated cytotoxicity, lysosome regulation of actin cytoskeleton and other pathways. These significantly enriched KEGG pathways reveal that molecular changes in high-risk group are closely related to the malignant characteristics of osteosarcoma, especially invasion and metastasis.

### 3.5. Analysis of Correlation between Risk Signature and Clinical Characteristics of Osteosarcoma

**Figure 5:**
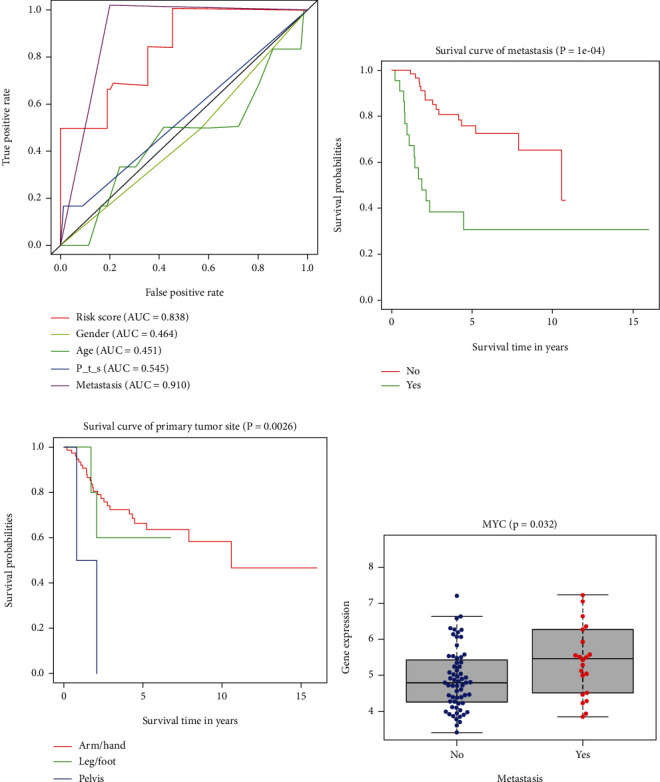
The relationship between risk signature and clinical characteristics. A, ROC curve was used to evaluate the effectiveness of riskScore and clinical characteristics in predicting OS. B-C, Kaplan-Meier survival curve shows the value of metastasis status andrimary tumor site in predicting the prognosis of patients with osteosarcoma. D, The relationship between OS-related ARGs and clinical characteristics. ROC: receiver operating characteristic; OS: overall survival; OS-related ARGs: autophagy-related genes related to overall survival; P_t_s: Primary tumor site.

In order to explore the relationship between risk signature and clinical characteristics, We first developed a multi-index ROC curve to compare the relationship between risk signature and clinical characteristics (gender, age, primary tumor site, and metastasis status) and prognosis (Figure 5(a)). The areas under the curve of riskScore, gender, age, primary tumor site and metastasis status are 0.838, 0.464, 0.451, 0.545 and 0.910, respectively. Subsequently, we analyzed the survival of four clinical characteristics to evaluate their value in predicting the prognosis of patients with osteosarcoma. Among them, the metastasis status (Figure 5(b), 5(p)<0.001) and the primary tumor site (Figure 5(C), 5P =0.0026) showed good predictive value. At the same time, we also analyzed the relationship between two OS-related ARGs screened by multivariate Cox regression and 4 clinical characteristics, and found that MYC was highly expressed in metastatic patients (Figure 5(D), 5P =0.032).

### 3.6. Development of a Nomogram for Predicting 1-, 3- and 5-Year Survival Rate of Osteosarcoma in Training Group

**Figure 6:**
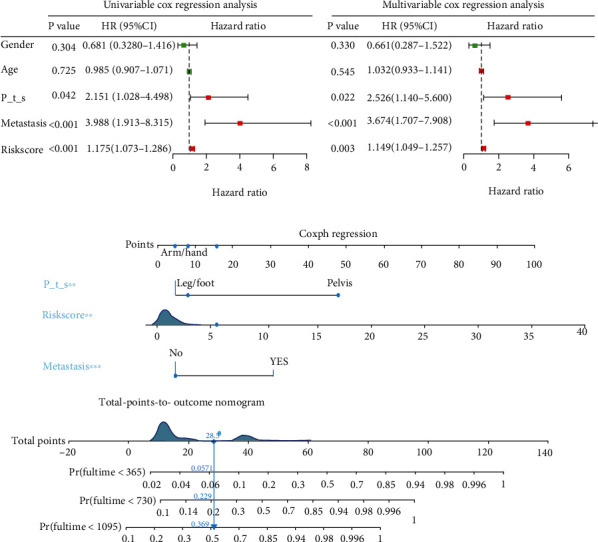
Construction of nomogram for predicting 1-, 3- and 5-year OS in patients with osteosarcoma. (a), Univariate and multivariate Cox regression analysis was used to evaluate the contribution of each variable to the OS of patients with osteosarcoma. (c), Establish a new type of nomogram. P_t_s: Primary tumor site; OS: overall survival.

**Figure 7:**
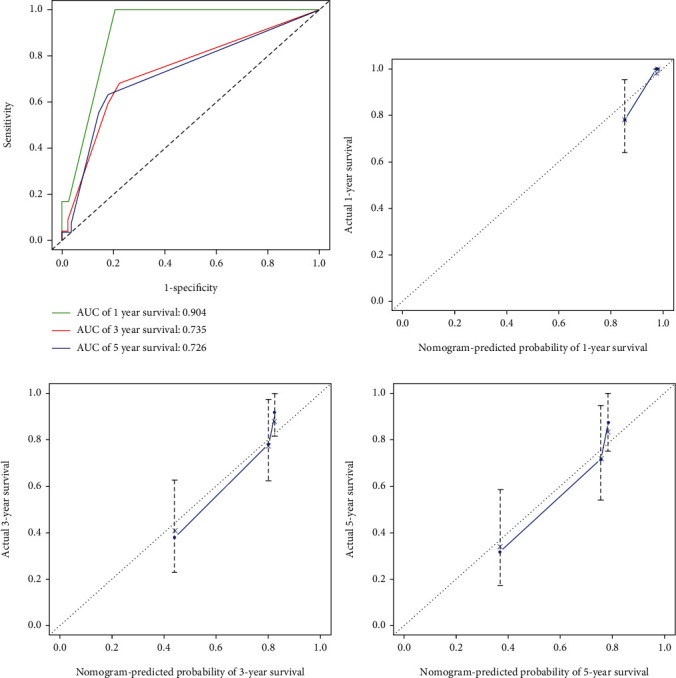
Evaluate the prediction efficiency of nomogram. A, ROC curve was used to evaluate the effectiveness of nomogram in predicting 1 -, 3-and 5-year OS. B-D, The calibration chart was used to evaluate the 1-, 3- and 5-year OS of nomogram. ROC: receiver operating characteristic; OS: overall survival.

In order to facilitate the clinical application of risk signature, we collected detailed clinical information of 85 patients with osteosarcoma from the TARGET database, including age, gender, primary tumor site, and metastasis status. First of all, univariate and multivariate Cox regression analysis of the collected data showed that riskScore, primary tumor site and metastasis status were independent risk factors for patients with osteosarcoma (Figure 6(a), 6P<0.05). Subsequently, a nomogram combining these three factors (Figure 6(b)) was constructed to predict the 1-, 3- and 5-year OS of osteosarcoma patients (estimated 1-, 3- and 5-year survival rates by adding up the scores of each factor). The performance of the nomogram is evaluated by C-index, ROC curve, and calibration chart. The C-index for predicting the OS of the model is 0.789 (95% CI: 0.703-0.875); ROC curve shows that the AUC for predicting 1-, 3- and 5-year survival rates are 0.904，0.735 and 0.726, respectively (Figure 7(a)). The results of C index and ROC curve show that the nomogram has a good degree of distinction. The calibration chart shows that the nomograph developed in this study has a good consistency between the prediction and observation of 1-, 3- and 5-year survival probabilities (Figures 7(b)-7(d)).

## 4. Discussion

Osteosarcoma is the most common primary malignant bone tumor in children, adolescents, and young people [22]. With the improvement of surgical techniques and the introduction of various treatment schemes, including chemotherapy and neoadjuvant chemotherapy, the cure rate has been improved, however, the effect is still not satisfactory for patients with metastatic or recurrent osteosarcoma, and the 5-year OS is only about 20% [7, 23]. In recent years, many studies have explored the role of autophagy in tumorigenesis and development, including in osteosarcoma, indicating that autophagy is closely related to the prognosis of osteosarcoma patients [11, 15]. However, most studies are focused on a single gene to study the relationship between autophagy and osteosarcoma [24–28], lacking a relatively systematic, multi-gene, and multi-angle analysis of the internal relationship between ARGs and osteosarcoma to reduce the single gene predict the existence of individual heterogeneity differences in the survival of osteosarcoma patients. In view of this, the purpose of this study is to extract ARGs related to the prognosis of patients with osteosarcoma through the TARGET database and construct a new autophagy-related prognosis model after comprehensive analysis combined with clinical characteristics.

In this study, we identified 16 OS-related ARGs of osteosarcoma by LASSO regression model. Considering that these genes may be related to the occurrence and development of osteosarcoma, we analyzed the GO and KEGG pathways of these genes. After the 16 OS-related ARGs were validated in the verification cohort and incorporated into multivariate Cox regression analysis, we finally screened 2 OS-related ARGs. According to these two genes, the riskScore of each patient is obtained, the risk signature are constructed, and the patients are further divided into high-risk group and low-risk group. Then, the risk signature were successfully verified in independent cohorts. Finally, we developed a nomogram which can be used to predict the prognosis of patients with osteosarcoma according to two clinical characteristics (primary tumor site, metastasis status) and riskScore, and confirmed the accuracy of nomogram prediction by C-index, ROC curve and calibration chart.

This study initially screened 16 ARGs that could be used to predict the survival of osteosarcoma patients by univariate Cox regression. GO and KEGG enrichment analysis showed that these genes were mainly involved in the regulation of autophagy. Studies have shown that autophagy is related to the occurrence, metastasis, apoptosis, and drug resistance of osteosarcoma [29–32]. Lorin et al. [33] found that the enhanced expression of damage-regulated autophagy modulator (DRAM) induced by 2-methoxyestradiol (2-ME) can promote autophagy and apoptosis of osteosarcoma. In addition, Mitophagy plays a role in mitochondrial quality control in mammalian cells and is crucial for the treatment of cancer and cancer off-target effects [34]. Studies have shown that norcantharidin (NCTD) can participate in inhibiting the growth of osteosarcoma through the mitophagy pathway [35]. These indicate that risk signature are closely related to autophagy and the regulation of autophagy, suggesting that risk signature may become a therapeutic target for patients with osteosarcoma. These findings are consistent with the results of this study.

In our study, two genes, ATG4A and MYC, were identified and verified as OS-related biomarkers, which were incorporated into the final prognostic signatures. According to these two OS-related ARGs, patients with osteosarcoma were divided into high-risk group and low-risk group. Kaplan-Meier analysis showed that the survival rate of the high-risk group was lower. In previous studies, the role of these genes in tumor prognosis has been widely reported. ATG4A is a member of the ATG4 family, and ATG4 is the site for the formation of autophagosome [36]. It is reported that ATG4A may be a biomarker of the prediction and prognosis of ovarian cancer [37]. MYC is a transcription factor located in the nucleus. MYC oncoproteins belong to the “hypertranscription factor” family and potentially regulate at least 15% of the transcription of the entire genome [38]. Chen et al. found that the high expression of MYC is related to poor survival, suggesting that MYC may be an important target for the treatment of osteosarcoma [39]. These results are basically consistent with our research results. In summary, almost all OS-related ARGs in risk signature are closely related to cancer. Therefore, it is reasonable to believe that these prognosis-related ARGs can be used as a reliable and reproducible prognostic biomarker in the future clinical application.

In order to better understand the relationship between these OS-related ARGs and the OS of patients with osteosarcoma. we further constructed the prognostic risk signature. Multi-index ROC curve analysis showed that the AUC (0.838) for predicting the risk signature of 1-year survival in patients with osteosarcoma was higher than that of gender (AUC =0.464), age (AUC =0.451) and primary tumor site (AUC =0.545). Indicating that the risk signature was more accurate for predicting the survival of osteosarcoma patients. Another innovation of this study is the development of a nomogram for clinical prediction of 1-, 3- and 5-year OS in patients with osteosarcoma, which combines riskScore, primary tumor site and metastasis status. C-index: 0.789 (95% CI: 0.703-0.875), ROC curve and calibration chart all verify the accuracy of nomogram prediction.

However, there are also some limitations in our research. First of all, the sample size of the TARGET database where the data of this study is located is limited. Secondly, treatment information cannot be obtained from the TARGET database, which may affect the prognosis of patients with osteosarcoma.. In addition, although the training cohort has been successfully verified in this study, a large number of external verification cohorts are still needed to verify its accuracy. In future research, we will collect more information and detailed sample data to overcome for these deficiencies.

## 5. Conclusions

To sum up, our study identified two OS-related ARGs related to the prognosis of patients with osteosarcoma. On this basis, a a risk profile was constructed and verified. GO and KEGG pathways enrichment analysis showed that the enrichment of risk signature was closely related to autophagy and the regulation. Besides, we developed a nomogram that combines risk signature with two clinical characteristics (primary tumor site, metastasis status), and proved that it has good accuracy in predicting 1-, 3- and 5-year OS in patients with osteosarcoma. However, further research is needed to verify the nomogram developed in our current research.

## Figures and Tables

**Table 1:** Demographic and Clinicopathological Features of Patients with Osteosarcoma.

Demographic or clinical characteristics	No. of samples	%
Gender		
Female	37	0.44
Male	48	0.56
Age		
≤15	49	0.58
>15	36	0.42
Race		
White	51	0.60
Asian	6	0.07
Black or African American	7	0.08
Unknown	21	0.25
Vital status		
Alive	47	0.55
Dead	38	0.45
Primary tumor site		
Leg/foot	77	0.07
Arm/hand	6	0.91
Pelvis	2	0.02
Metastasis status		
No	63	0.74
Yes	22	0.26

## Data Availability

The analyzed datasets generated during the study are available from the corresponding authors on reasonable request.

## Supplementary Materials

Supplementary 1 Supplementary Figure S1: LASSO analysis of ARGs related to OS in the training cohort.

Supplementary 2 Supplementary Table S1: Demographic and Clinicopathological Features of Patients with Osteosarcoma in verification group.

Supplementary 3 Supplementary file 1: Univariate Cox regression screening for OS-related ARGs.

Supplementary 4 Supplementary file 2: The results of LASSO regression analysis.

Supplementary 5 Supplementary file 3: The four OS- related ARGs successfully verified.

Supplementary 6 Supplementary file 4: Multivariate Cox regression analysis results.
