# Supplementary figures and images for: Establishment of an Autophagy-Related Clinical Prognosis Model for Predicting the Overall Survival of Osteosarcoma
Source: Biomed Res Int. 2021 Sep 22;2021:5428425. doi: 10.1155/2021/5428425 (PMC8485141; doi:10.1155/2021/5428425)

**A**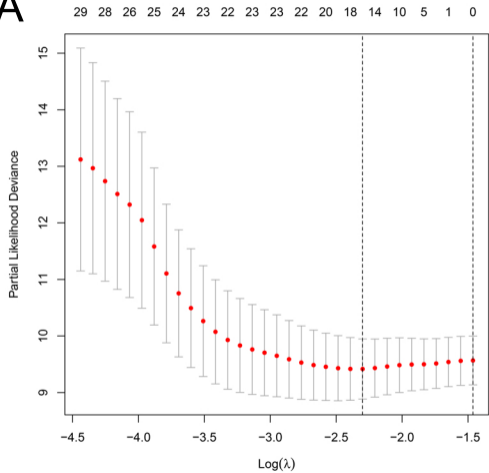**B**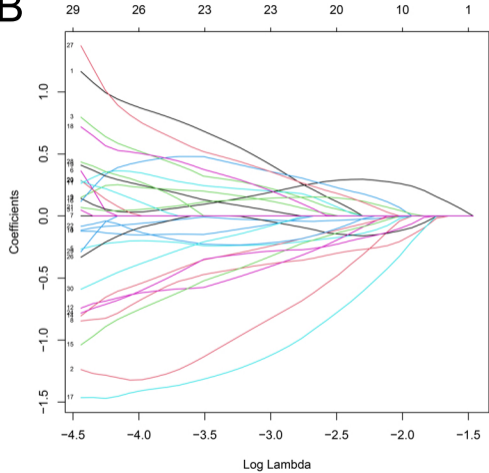

Supplement: Supplementary 1 — Supplementary Figure S1: LASSO analysis of ARGs related to OS in the training cohort. [file 5428425.f1.pdf]
