# Supplementary material for: Establishment of an Autophagy-Related Clinical Prognosis Model for Predicting the Overall Survival of Osteosarcoma
Source: Biomed Res Int. 2021 Sep 22;2021:5428425. doi: 10.1155/2021/5428425 (PMC8485141; doi:10.1155/2021/5428425)
Supplement: Supplementary 2 — Supplementary Table S1: Demographic and Clinicopathological Features of Patients with Osteosarcoma in verification group. [file 5428425.f2.docx]

| Table S1. Demographic and Clinicopathological Features of Patients with Osteosarcoma in verification group | | |
| --- | --- | --- |
| Demographic or Clinical characteristics | No. of Samples | % |
| Gender |  |  |
| Female | 19 | 0.36 |
| Male | 34 | 0.64 |
| Age |  |  |
| ≤15 | 19 | 0.36 |
| >15 | 34 | 0.64 |
| Vital Status |  |  |
| Alive | 30 | 0.57 |
| Dead | 23 | 0.43 |
| Primary tumor site |  |  |
| Leg/Foot | 44 | 0.83 |
| Arm/Hand | 8 | 0.15 |
| Unknown | 1 | 0.02 |
| Metastasis status |  |  |
| No | 19 | 0.36 |
| Yes | 34 | 0.64 |
